# Efficacy of Extracorporeal Shock Wave Therapy for Lateral Epicondylitis: A Systematic Review and Meta-Analysis

**DOI:** 10.1155/2020/2064781

**Published:** 2020-03-18

**Authors:** Gaowen Yao, Jing Chen, Yanji Duan, Xiao Chen

**Affiliations:** ^1^The Department of Orthopedic Surgery, The First People's Hospital of Neijiang, Neijiang, 641000 Sichuan Province, China; ^2^The Department of Neonatology, The First People's Hospital of Neijiang, Neijiang, 641000 Sichuan Province, China

## Abstract

**Background:**

Lateral epicondylitis (LE) is a common elbow problem. Extracorporeal shock wave therapy (ESWT) was widely used in the treatment of LE and has been shown to relieve the pain and functional impairment (loss of grip strength) caused by tennis elbow. However, the evidence with regard to whether ESWT has better clinical efficacy over other method is not clear. The aim of the study was to compare the effectiveness of ESWT with other techniques in the treatment of LE.

**Methods:**

Literature searches of PubMed, OVID, Embase, Cochrane Library, and Web of Science were searched up to 30^th^ June, 2019. Only RCTs comparing ESWT with other methods for LE were included. Data collection and extraction, quality assessment, and data analyses were performed according to the Cochrane standards.

**Results:**

A total of 13 articles with 1035 patients were included. Of which, 501 underwent ESWT and 534 underwent other methods. The result of meta-analysis showed that pooled VAS (*P* = 0.0004) and grip strength (*P* < 0.00001) were better in the ESWT group.

**Conclusion:**

Based on the existing clinical evidence, extracorporeal shock wave therapy can effectively relieve the pain and functional impairment (loss of grip strength) caused by tennis elbow, with better overall safety than several other methods. However, owing to the limited quality and quantity of the included studies, more high-quality RCTs are needed to support the trend towards better functional outcomes with ESWT.

## 1. Introduction

Lateral epicondylitis (LE) is a common musculoskeletal pathology arising secondary to recurrent microtrauma of the upper extremity, particularly impacting the lateral epicondyle of the elbow [1, 2]. Major symptoms include decreased grip and upper-extremity strength along with pain and inflammation originating from the lateral elbow. Annual incidence is 1%–3% of the global population [3], with people aged 35 years and older being the most frequently afflicted [4–6]. There is little difference in prevalence between males and females. It is more frequent in the dominant arm, consistent with overuse as a major causative factor. Lateral epicondylitis is also known as “tennis elbow” as it is observed with particularly high frequency (5%–10%) in tennis players [7].

Several approaches have been used for the treatment of LE, such as physical therapy (including rest and movement restriction, activity modification, hot−cold application, electrotherapy, massage, and ultrasound), splinting, local injections (corticosteroids and platelet-rich plasma), oral or topical nonsteroidal anti-inflammatory drugs, and surgery [8, 9]. Treatments are mainly aimed at reducing pain, controlling inflammation, accelerating healing, and ensuring that the patient can perform activities of daily life. However, there is insufficient evidence to support the general efficacy of these treatments [10, 11]. Further, the side effects of anti-inflammatory drugs are a major concern, especially for the elderly or people with comorbid diseases sensitive to immunosuppression [12]. Further, pain is actually aggravated when cortisone injection is first started, and the risk of recurrence remains elevated despite relief of pain and inflammation [13].

Extracorporeal shock wave therapy (ESWT) is a noninvasive procedure in which acoustic waves are focused on targeted sites within the body to facilitate pain relief and healing [14]. In general, ESWT is considered safe, noninvasive, easy to apply, and well tolerated by most patients, and so has been widely used for many musculoskeletal conditions over the last 25–30 years [15]. Although the exact mechanisms for analgesic and functional effects are still incompletely understood, it is suggested that shock waves accelerate tissue regeneration, reduce calcification, and inhibit pain receptors [16].

However, the efficacy of ESWT for tennis elbow remains controversial, as some studies have reported that ESWT is highly effective and even a reasonable alternative to surgery, while others have reported effects not markedly different from placebo [17–19]. To provide support for clinical decisions, we conducted a systematic review and meta-analysis of currently available prospective randomized controlled trials evaluating the effectiveness and safety of ESWT for the treatment of LE.

## 2. Materials and Methods

We followed the methods of our previously published manuscript at 2018 [20].

### 2.1. Data Sources and Searches

The following electronic databases, including PubMed (1966 to April 2019), Embase (1974 to April 2019), the Cochrane Library (April 2019), Web of Science (1990 to April 2019), OVID (April 2019), CBM (April 2019), Wan Fang (April 2019), and China National Knowledge Infrastructure (April 2019), were searched. To search more potentially eligible studies, the Google Search Engine was also used ending up to April 2019. In addition, reference lists of identified reports were reviewed for other potentially relevant studies. The following keywords were used to search the databases: “lateral epicondylitis,” “lateral epicondylitides,” “lateral humeral epicondylitis,” “lateral humeral epicondylitides,” “tennis elbow,” “tennis elbows,” “extracorporeal shock wave therapy,” “ESWT,” “shock wave,” “high-energy shock wave,” “high energy shock waves,” “ultrasonic shock wave,” “ultrasonic shock waves,” “ultrasonic shockwave,” “ultrasonic shockwaves,” and “randomized controlled trial.” The search was limited to randomized controlled trials (RCTs) published in English.

### 2.2. Study Selection

Studies were selected based on the following inclusion criteria: (1) study design: prospective randomized controlled trial, (2) patients: patients diagnosed with later epicondylitis, (3) intervention: extracorporeal shock wave therapy vs. control or other methods, and (4) outcome: visual analogue scale (VAS) and grip strength. Exclusion criteria were as follows: (1) retrospective studies, animal studies, single-case reports, protocols, reviews, meta-analyses, poster presentations, or conference abstracts; (2) study objective or intervention measures failed to meet the inclusion criteria; (3) duplicate or multiple publications of the same study; and (4) studies without usable data.

### 2.3. Data Extraction and Quality Assessment

The abstracts of retrieved studies were independently reviewed by two authors, and full articles were examined when necessary. The data were extracted independently by these two authors, and any disagreements were resolved by discussion with at least one more author until a consensus was reached. If more than one article was published from the same cohort, the study with the most comprehensive data was selected for inclusion.

The following information was extracted from all qualifying articles: general information (name of first author, publication year, region where the population resided, study type, sample size, mean ages, and interventions) and outcomes (as defined above).

The quality of the included trials was assessed independently by two reviewers using the Cochrane Risk of Bias Tool of Review Manager Version 5.3. Appraisal criteria included random sequence generation, allocation concealment, blinding of participants and personnel, blinding of outcome assessment, incomplete outcome data, selective reporting, and other sources of bias. Each of these factors was recorded as high risk, low risk, or unclear risk. If data were unclear, we contacted study authors for clarification whenever possible.

### 2.4. Statistical Analysis

The extracted data were pooled using Review Manager 5.3. Risk ratios (RRs) were calculated for dichotomous variables in each study. Weighted mean differences (WMDs) were calculated for continuous variables, and 95% confidence intervals (CIs) were calculated for all effect sizes. Cochran's *Q* test and Higgins *I*^2^ statistic were used to measure the heterogeneity of the included studies. An *I*^2^ ≥ 50% or *P* < 0.05 was considered significant heterogeneity. In such cases, the random effects model was applied; otherwise, a fixed effect model was used. Potential publication bias was assessed by Begg's and Egger's tests. Sensitivity analysis was applied to evaluate the robustness of results. A *P* < 0.05 was defined as statistically significant for all tests.

## 3. Results

### 3.1. Search Results

**Figure 1:**
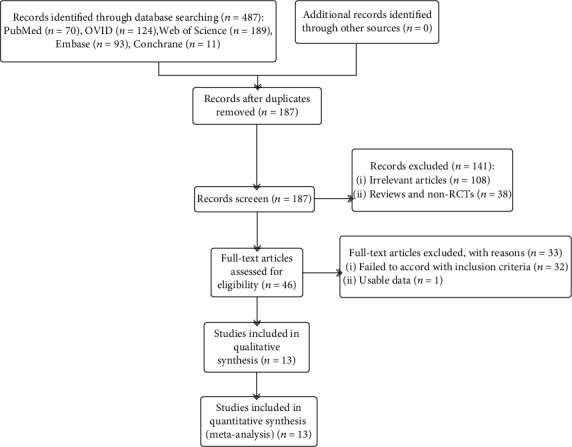
Flow chart of the literature selection.

Figure 1 illustrates the detailed steps of literature selection. A total of 187 potentially relevant articles were obtained after exclusion of duplicates. After further review of the titles and/or abstracts, 141 studies were excluded as retrospective studies, animal studies, single-case reports, protocols, reviews, meta-analyses, poster presentations, conference abstracts, or articles without usable data. After reading the full text of the remaining 14 articles, one trial was identified that did not provide standard deviations (SDs) and so was also excluded. Finally, 13 articles describing the results of RCTs with a total of 1035 patients were included in the meta-analysis. The main characteristics of these studies and patients are summarized in Table 1.

### 3.2. Quality Assessment and Basic Information

**Figure 2:**
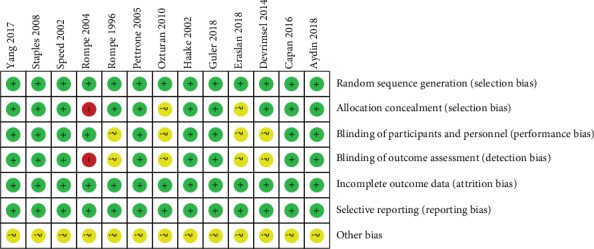
Risk of bias assessment summary.

According to the Cochrane Collaboration Risk of Bias Tool, the quality of all RCTs was acceptable (Figure 2). Thirteen RCTs reported the method of randomization, with 10 randomized through computer-generated lists and 3 through random numbers. Blinding was maintained using sealed envelopes in 10 studies, and 9 reported blinding of the operator and participants. No study showed an unclear bias due to incomplete outcome data or selective outcome reporting.

### 3.3. Meta-Analysis of Pain Evaluation

**Figure 3:**
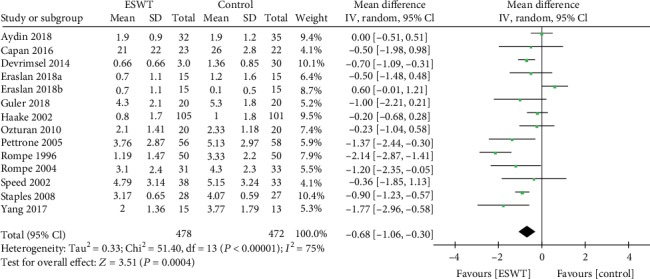
Forest plot of differences in visual analogue score between ESWT and control groups.

Thirteen articles describing 14 trials with a total of 950 patients reported relevant data regarding posttreatment pain evaluation by VAS. There was significant heterogeneity observed among studies (*I*^2^ = 75%, *P* < 0.00001); therefore, a random effects model was used. The pooled data showed significantly lower VAS in the ESWT group (WMD = −0.68; 95%CI = −1.06 to − 0.30, *P* = 0.0004; Figure 3).

### 3.4. Meta-Analysis of Grip Strength

**Figure 4:**
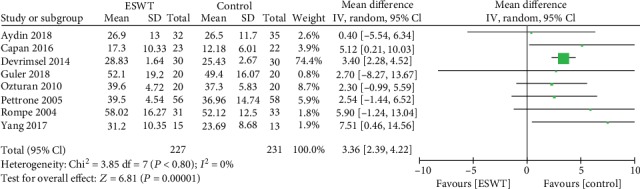
Forest plot of grip strength differences between ESWT and control groups.

Eight articles with 458 patients provide data on grip strength. There was no significant heterogeneity observed among studies (*I*^2^ = 0%, *P* = 0.80); therefore, a fixed effects model was used. The meta-analysis showed that the patients in the ESWT group significantly increased the grip strength (WMD = 3.36; 95%CI = 2.39 to 4.33, *P* < 0.00001; Figure 4).

### 3.5. Publication Bias

**Figure 5:**
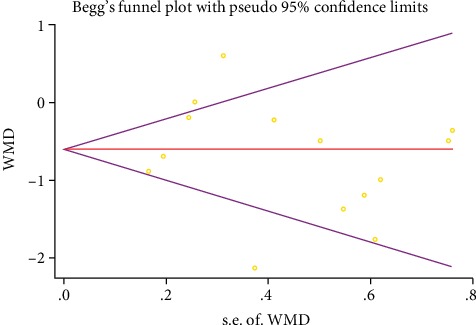
Funnel plot of Begg's test for VAS publication bias (WMD, weighted mean difference).

**Figure 6:**
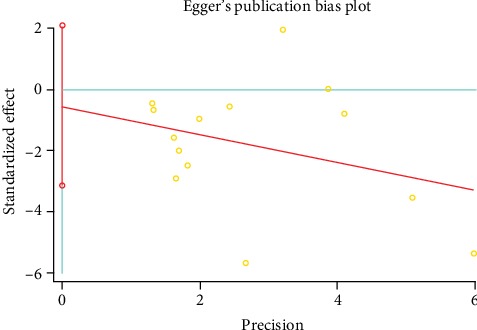
Egger's publication bias plot for the VAS.

No significant publication bias was found for VAS (Figures 5 and 6). Test results also confirmed that the potential publication bias was negative (*P*_Begg's test_ = 0.913; *P*_Egger's test_ = 0.656).

### 3.6. Sensitivity Analysis

**Figure 7:**
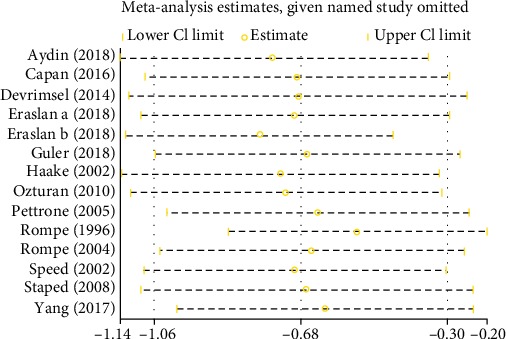
Sensitivity analysis of VAS.

Sensitivity analysis was conducted to assess the potential impact of each individual study on the pooled results. As illustrated in Figure 7, there was relative consistency among studies with no major outliers.

## 4. Discussion

The symptoms of tennis elbow (lateral epicondylitis) can persist for between 6 months and 2 years, although the condition usually resolves within 1 year with appropriate care (e.g., rest and load restriction) [32]. However, 4%–11% of patients require surgical intervention, and many patients with severe functional impairment or pain require other nonsurgical treatments [26]. Presently, ESWT is only a secondary option for LE treatment and an alternative for patients who refuse surgical treatment.

The main impairments of LE are pain, decreased grip strength, and impaired upper-extremity strength. In the majority of studies, the VAS was used to evaluate pain at rest or during activity (0 indicating no pain and 10 the worst pain). Grip strength is a useful objective measure for evaluating disease severity, treatment response, and functional recovery. In most studies, grip strength was measured using a Jamar hand dynamometer equipped with an analogue display (up to 200 lb. or 90 kg) [15]. Therefore, we used VAS and grip strength as the primary outcomes to evaluate the clinical efficacy of ESWT for LE treatment in this meta-analysis, and only included randomized controlled trials to ensure the reliability of the pooled results.

The main goals of therapy are to control pain and maintain or improve function. Once pain is controlled, function may be improved through exercise to strengthen the elbow joint and extend the range of motion [33]. According to pooled results, early recovery was accelerated by ESWT as measured by VAS (*P* = 0.0004) and grip strength (*P* < 0.00001). Rompe et al. evaluated and compared the therapeutic effects of ESWT to placebo among patients with tennis elbow receiving no conservative treatment for the previous 6 months or more. They found significant alleviation of pain and improvement of function in the ESWT group, with good or excellent outcome in 48% and acceptable results in 42% at the final review compared to only 6% and 24%, respectively, in the placebo group [24]. Pettrone and McCall [25] examined the therapeutic effect of ESWT on tennis elbow patients who had experienced at least two conservative treatment failures in a multicenter, randomized, placebo-controlled study. The results showed significant improvements in pain score, functional activity score, specific activity evaluations, and overall evaluations of disease state in the ESWT group compared to the control group. Therefore, ESWT may be a good choice for tennis elbow when conventional treatments are ineffective. Similarly, Devrimsel et al. [28] reported that ESWT was significantly better than laser therapy for LE treatment, and Vulpiani et al. [34] found that ESWT was superior to cryoultrasound at 6 months and 1 year posttreatment according to VAS scores and patient satisfaction ratings.

However, not all studies have found clear evidence for the superiority of ESWT over other treatments or placebo. A multicenter, randomized, placebo-controlled single-blind study by Haake et al. found that ESWT under local anesthesia was no more effective than placebo at 12 weeks (25.8% vs. 25.4% success rates) [22]. Similarly, Lee et al. [35] found that ESWT was no more effective than corticosteroid injection for newly diagnosed LE and medical epicondylitis immediately after treatment and at 8-week follow-up. However, longer term follow-up may reveal differences not observed in the early posttreatment stage. Ozturan et al. [27] compared clinical outcomes over 52 weeks among patients with >6 months disease history receiving ESWT, autologous blood injection, or corticosteroid. Evaluations using VAS and grip strength revealed superior outcomes in the corticosteroid group at 4 weeks, while at 52 weeks, the success rate of corticosteroid injection (50%) was substantially lower than autologous blood injection (83%) and ESWT (89%). Eraslan et al. [30] and Aydin and Atic [1] drew similar conclusions. Thus, ESWT appears to demonstrate greater long-term efficacy than corticosteroid injection.

These disparate outcomes may be explained by methodological differences among studies, including the number of ESWT pulses delivered, pulse frequency, duration of application, follow-up duration, treatment interval, specific devices used, and evaluation methods. Nonetheless, as there is still controversy regarding the efficacy of ESWT for LE, we conducted this meta-analysis of good quality standardized, prospective, randomized, single- or double-blind studies evaluating ESWT using pain VAS and grip strength as outcome measures. Indeed, pooled results revealed that most LE patients reported better and faster pain reduction and demonstrated superior long-term grip strength improvement after ESWT compared to other therapies.

At present, the mechanisms of shock wave therapy for tennis elbow are not completely clear. Yang et al. [15] found that cracks in the extensor tendon were significantly reduced and that muscle tendon cracks were undetectable in all shock wave group patients according to 2D ultrasonic examination before studying the knot bundle. They speculated that ESWT can induce fibroblast reactions that gradually heal cracks in the extensor tendon. In addition, shock wave has been shown to induce angiogenesis by stimulating proangiogenic factors, thereby increasing blood flow, promoting regeneration of tissues, and reducing pain [36, 37]. These waves dissipate energy at the interface of 2 substances (tissues) with different acoustic impedance values, such as the bone−tendon interface, resulting in the release of kinetic energy that can trigger tissue reparative processes. In addition, it has been hypothesized that ESWT works by stimulating nerve fibers to produce analgesia and that disruption of the tendon tissue may induce healing. Other studies have shown that shock waves can reduce the expression of calcitonin-related peptide in dorsal root ganglia, thus relieving pain [38].

The following limitations of this meta-analysis should be acknowledged. First, shock wave instruments differed among studies, which may have contributed to the variation in outcome measures. Second, there have been relatively few studies on the optimal therapeutic shock wave protocol for tennis elbow, which likely contributed to the variable efficacy reported in the included studies. Third, we included RCTs from different countries and with different diagnostic criteria, which also introduced substantial clinical heterogeneity. Fourth, almost half of the studies included were conducted in one smaller country, Turkey, which may reduce the generalizability of our conclusions. Nonetheless, included studies were also conducted in America, Europe, and Asia.

In conclusion, based on the existing clinical evidence, extracorporeal shock wave therapy can effectively relieve the pain and functional impairment (loss of grip strength) caused by tennis elbow, with better overall safety than several other methods, especially corticosteroid injection. For patients unresponsive to conventional treatment methods, clinicians should consider shock wave therapy as a possible alternative to reduce pain and medical expenses associated with more invasive therapy. Although it is still necessary to identify the mechanisms of ESWT and the optimal application parameters, there is compelling evidence that ESWT can be used as a conventional noninvasive alternative for treating tennis elbow. Additional high-quality RCTs are required to verify and supplement these conclusions.

## Figures and Tables

**Table 1:** Summary of study and patient characteristics.

Study (year)	Country	Subjects per intervention	Intervention	Age (year)	M/F	Medical history	Outcome	Pulses	Energy density/pressure
Rompe [21] (1996)	Germany	50/50	ESWT Placebo	43.9 41.9	42/58	24.8 m 21.9 m	VAS	1000	0.08 mJ/mm^2^
Haake [22] (2002)	Germany	134/137	ESWT Placebo	46.9 46.3	128/143	27.6 m 22.8 m	VAS	2000	0.07-0.09 mJ/mm^2^
Speed [23] (2002)	England	40/35	ESWT Placebo	46.5 48.2	33/42	15.9 m 12 m	VAS	1500	0.12 mJ/mm^2^
Rompe [24] (2004)	Germany	34/36	ESWT Placebo	45.9 46.2	36/34	>12 m	VAS, grip strength	2000	0.09 mJ/mm^2^
Pettrone [25] (2005)	America	56/58	ESWT Placebo	>18 >18	—	>6 m	VAS, grip strength	2000	0.06 mJ/mm^2^
Staples [26] (2008)	America	33/30	ESWT Placebo	49.8 49.1	37/26	52.6w 68w	VAS	2000	0.03 mJ/mm^2^
Ozturan [27] (2010)	Turkey	19/18/20	ESWT/ABI/CSI	47/44/45.8	25/32	9.6 m/10 m/9.5 m	VAS, grip strength	2000	0.17 mJ/mm^2^
Devrimsel [28] (2014)	Turkey	30/30	ESWT Laser	37.76 40.30	—	14.16 m 13.43 m	VAS, grip strength	—	—
Capan [29] (2016)	Turkey	23/22	ESWT Placebo	48.4 46.2	10/35	7.9 m 7.7 m	VAS, grip strength	2000	1.8 bar
Yang [15] (2017)	China	15/13	ESWT Physiotherapy	50.93 51.08	12/16	6.53 m 7.31 m	VAS, grip strength	2000	0.7 mJ/mm^2^
Aydin [1] (2018)	Turkey	32/35	ESWT WESs	38.84 37.94	34/33	—	VAS, grip strength	2000	1.6-1.8 bar
Eraslan [30] (2018)	Turkey	15/15/15	ESWT/physiotherapy/kinesiotaping	48/47.2/48.5	—	—	VAS	2000	0.06-0.12 mJ/mm^2^
Guler [31] (2018)	Turkey	20/20	ESWT Placebo	46.3/45.8	12/28	>3 m	VAS, grip strength	1500	2.4 bar

ESWT: extracorporeal shock wave therapy; ABI: autologous blood injection; CSI: corticosteroid injection; WESs: wrist-extensor splints; VAS: visual analogue scale.
